# P-2146. Comparing Valganciclovir Exposures to Predict Neutropenia in Pediatric Solid Organ Transplant Recipients

**DOI:** 10.1093/ofid/ofaf695.2309

**Published:** 2026-01-11

**Authors:** Mai Uyen Nguyen, Michael N Neely, Anders Åsberg, Kevin J Downes

**Affiliations:** Thomas Jefferson University, Children's Hospital of Philadelphia, Philadelphia, PA; Children’s Hospital Los Angeles, University of Southern California, Los Angeles, CA, USA, Los Angeles, CA; University of Oslo, Oslo, Oslo, Norway; Children's Hospital of Philadelphia, Philadelphia, PA

## Abstract

**Background:**

Valganciclovir (VGCV) is an antiviral commonly used for cytomegalovirus (CMV) prevention in pediatric solid organ transplant (pSOT) recipients. Neutropenia is a common toxicity, but therapeutic drug monitoring (TDM) is not routinely available. To evaluate the exposure-toxicity relationship, we compared simulated ganciclovir (GCV) exposures among pSOT recipients with and without neutropenia during VGCV prophylaxis.

**Figure 1:**
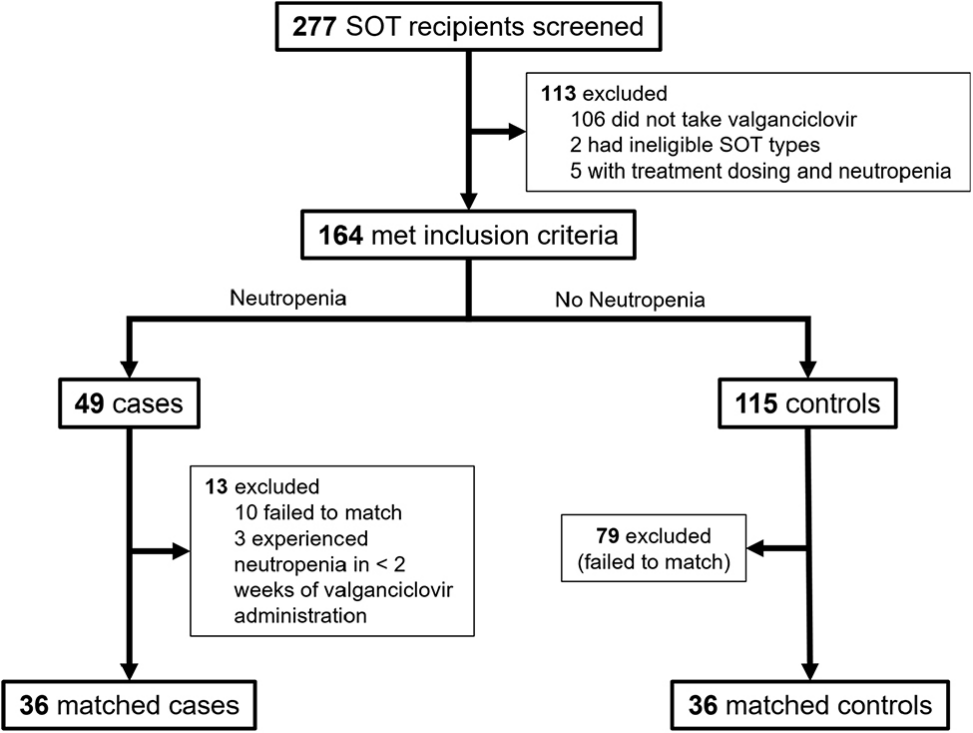
Flowchart for subject eligibility and matching. Neutropenia is defined as an ANC measurement of < 1,000/µL after at least 2 weeks of valganciclovir prophylaxis but also have any preceding ANC value of at least 1,500/µL.

**Table 1:**
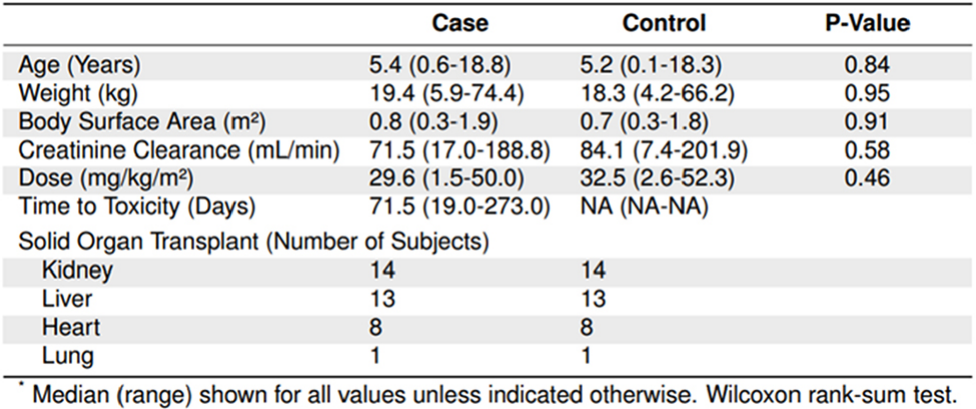
Baseline characteristics of matched case and controls at the start of valganciclovir treatment. Creatinine clearance was calculated using the Cockcroft-Gault equation. Body surface area was calculated using the Mosteller formula.

**Methods:**

We performed a retrospective matched case-control study among pSOT recipients at the Children’s Hospital of Philadelphia; body surface area-based VGCV dosing was used in all children. Cases were defined as an absolute neutrophil count (ANC) < 1,000/µL while taking VGCV for ≥ 2 weeks and had at least 1 preceding ANC value of > 1,500/µL. Controls were children without neutropenia matched to a case by age (+/- 1 year), organ, and duration of VGCV prophylaxis. We used a published population pharmacokinetic model (Åsberg et al, 2014) to simulate GCV concentrations with Pmetrics, accounting for each subject’s time-dependent variables (age, weight, creatinine clearance). We then calculated 24-hour, 7-day, and cumulative area under the curve (AUC) in each subject and used conditional logistic regression to compare GCV exposures among cases and controls.

**Figure 2:**
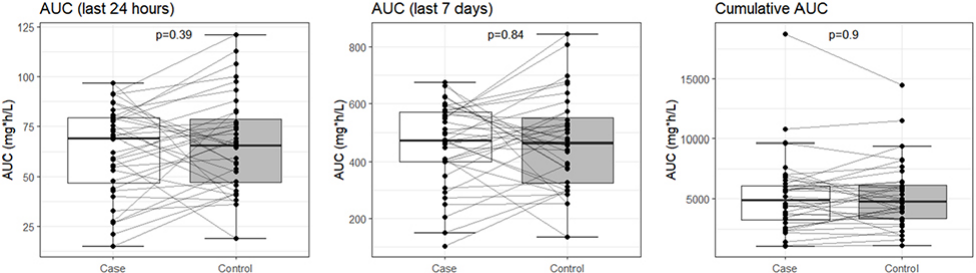
Valganciclovir exposures do not differ between cases and controls. AUC of the case and control groups for the last 24 hours (left), last 7 days (middle), and entire treatment duration (cumulative, right) before neutropenia. AUCs are derived from simulated GCV concentrations. Each point represents an individual subject, which is connected to their matched case or control with a gray line. P-values were calculated with conditional logistic regression.

**Figure 3:**
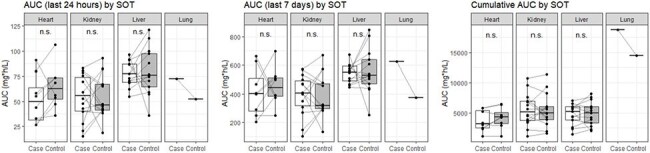
Valganciclovir exposures among cases and controls categorized by solid organ transplant (SOT) type. AUC of the case and control groups for the last 24 hours (left), last 7 days (middle), and entire treatment duration (cumulative, right) before neutropenia categorized by each SOT. AUCs are derived from simulated GCV concentrations. Each point represents an individual subject, which is connected to their matched case or control with a gray line. All p-values > 0.05 for each SOT type and each timeframe (conditional logistic regression).

**Results:**

Among a cohort of 277 pSOT recipients, we identified 36 cases and 36 matched controls (Figure 1). Case-control matches had similar baseline characteristics at the start of VGCV therapy (Table 1). There were no statistically significant differences in the 24-hour [odds ratio (OR) 0.988, 95% confidence interval (CI) 0.961-1.015], 7-day (OR 0.9996, 95% CI 0.996-1.003), or cumulative AUCs (OR 1.00, 95% CI 0.9996-1.00) among all cases and controls (Figure 2). AUC metrics by SOT type also showed no statistically significant differences (Figure 3).

**Conclusion:**

Simulated GCV exposures were similar among pSOT recipients with and without neutropenia in our study, suggesting that differences in dosing and covariates did not drive toxicity in our population. Future studies should directly measure GCV concentrations to discern whether toxicity relates to exposures/concentrations or intrinsic factors (i.e. genetics) in the pSOT population.

**Disclosures:**

Kevin J. Downes, MD, Paratek Pharmaceuticals, Inc.: Grant/Research Support|Veloxis Pharmaceuticals, Inc.: Grant/Research Support

